# An Eighteenth Century Tuberculosis Conceit

**Published:** 1904-12

**Authors:** 


### EDITORIAL NOTES AND COMMENTS.

The Business office of THE JOURNAL-RECORD is No. 1007-1008 Century Building.

The Editorial office is 318-319-320 The Prudential.

Address all Business communications to Dr. M. B. Hutchins, Mgr.

Make remittances payable to THE ATLANTA JOURNAL-RECORD OF MEDICINE.

On matters pertaining to the Editorial and Original Communications address Dr. Bernard Wolff, Atlanta.

Reprints of original articles will be furnished at cost price. Requests for the same should always be made *on the manuscript*.

We will present, post-paid, on request, to each contributor of an original article, twenty (20) marked copies of THE JOURNAL-RECORD containing such article.

### AN EIGHTEENTH CENTURY TUBERCULOSIS CONCEIT.

THE following quotation forms a part of a letter to a popular magazine which has joined effectively in the anti-tuberculosis crusade and is the production of a doctor in Tennessee :

The theory of contagion is based upon the propagation of the tubercular bacillus, and is more of a theological conception than a biological one. It is a mistake that the evolvement of life-cells ceased upon this planet untold years ago. It is still in progress, but these lower forms are almost as yet beyond our means of observation. Tubercles have often been found with no bacilli in them. Bacilli are a product of the decomposition of the tubercle rather than its cause. Tubercles are a product of imperfect combustion of waste albuminoid substances, and this imperfect combustion is the basis of that want of assimilation which precedes the deposition of tubercles in every case of consumption. No one advocates the doctrine that consumption is directly contagious, for there are too many exposures without contracting the disease for this to be tenable; but of late years it is claimed that the sputum becomes desiccated and dried and the bacillus floats on "airy wings" in search of a victim. This, to say the least, is far-fetched. When the protoplasm of a cell once becomes dried that is the end of its life principle.

This quotation is inserted as an illustration of what obstacles anti-tuberculosis movements have to encounter, even within the medical profession, and as offering a singularly complete exhibition of ignorance and archaic misconception. There is not a correct sentence in it, nor one that is remotely true. Ignorance of elementary pathology shows itself even in the misuse of the adjective tubercular. A tubercular bacillus is a bacillus that is affected with tuberculosis. Would that it were possible for this ubiquitous germ to languish with the same malady that it engenders. Alas! the methods of diagnostic precision have not been drawn to so fine a point as to establish the disease in the germ itself, else it would be a noble triumph of science to be able to inject a few drops of tuberculin in the lumbar region of T. B. to observe the specific reaction before turning him out on the public. But this, as the doctor elsewhere remarks, is merely thrown out as food for thought.

It is not apparent how the theory of the contagion of consumption is a theological rather than a biological conception unless one stops to consider how many mortals have put on immortality and gone to join the angels from failure to embrace the theory of contagion.

Those who follow modern rules and methods and not those that might have been gotten from the the book of Leviticus, find the problem intensely biological. It is true that we have no means of observing these low forms of life, and yet for some years there have been those who were more or less familiar with an instrument for magnifying objects called the microscope and who are able to observe and say a few words about quite a number of these low forms of life. Was not the existence of the tubercle bacillus established in this way, for even our theological doctor must acknowledge its existence, otherwise he would find himself animadverting against a thing that was non-existent, which is almost as absurd as his present position.

"Bacilli are the product of the decomposition of the tubercle rather than its cause." *Omne vivum ex vivo* is a liberal rendering of Virchow's dictum, and if there be no life in the tubercle how may life proceed from it? The doctor perhaps had in mind the riddle of Samson, where the bees and honey were taken from the carcass of the lion—rendering the proposition theological or Biblical rather than biological. We pass over the description of the process of tubercle formation as too profound for flippant discussion, but the next sentence flies like a cinder in the eye. Has the doctor rotted in ease in the solitude of the Cumberland plateau that no whisper has reached him of the communicable nature of tuberculosis? It is scarcely credible that no rumor to this effect has penetrated the cloistral seclusion of the doctor. The reverse of the proposition might be stated as a truism that those who do not believe in the contagious nature of consumption might hold an indignation meeting in the preparation room of the Odd Fellows' Hall in the doctor's native village and then there would be present no more than a *cujus.*

The far-fetched theory of dried bacilli floating on "airy wings" (a poetic phrase only, for all the world knows that wings are not airy—they merely need air to flop in) in search of a victim is, indeed, overstrained. Who ever heard of a dried seed sprouting, or who ever saw a cake of Fleischmann's yeast that would make bread rise? Of course, the life of the protoplasm takes its flight in the process of desiccation. "If these things are true, why should they lie hid?" to quote Sir Toby Belch.

But these unique and ancient views are not merely diverting, they are pernicious. The magazine containing the doctor's communication (and a correspondent of the magazine very properly makes a rebuttal) has a circulation that reaches the half-million mark. More laymen read it than there are doctors in the Western hemisphere and some are impressed by it and set back a century in the march of time. The protection of society imperatively demands a clear conception of the nature of its worst enemy in the form of disease, and it is just such well-meaning but misguided persons like this Tennessee doctor that break through the phalanx and speed the shafts of death. The effect upon the public is deeper when such views are delivered by a medical man and therefore harder to correct. Of course, the day will come when consumption will be known of the people as it should be, but the obstructions to this knowledge are heavy and to be removed only by infinite labor.

